# The Validation of an Experimental Model in Wistar Female Rats to Study Osteopenia and Osteoporosis

**DOI:** 10.3390/bioengineering12070702

**Published:** 2025-06-27

**Authors:** Artur Lage Pedroso, Raul Canal, Sergio Alexandre Gehrke, Eleani Maria da Costa, Antonio Scarano, Fernanda Barchesi Zanelatto, André Antonio Pelegrine

**Affiliations:** 1 Division of Implant Dentistry, Faculdade São Leopoldo Mandic, Campinas 13045-755, Brazil; artur.pedroso@prof.una.br (A.L.P.); fernanda.zanelatto@slmandic.edu.br (F.B.Z.); 2 Sociedade Brasileira de Direito Médico e Bioética, Brasília 70322-901, Brazil; presidencia@anadem.org.br; 3 Department of Implantology, Bioface/Postgrados en Odontología/Universidad Catolica de Murcia, Montevideo 11100, Uruguay; 4 Department of Biotechnology, Universidad Católica de Murcia, 30107 Murcia, Spain; 5 Department of Materials Engineering, Pontificial Catholic University of Rio Grande do Sul, Porto Alegre 90619-900, Brazil; eleani@pucrs.br; 6 Department of Innovative Technologies in Medicine & Dentistry, University of Chieti-Pescara, 66013 Chieti, Italy; ascarano@unich.it

**Keywords:** animal study, bone density, female Wistar rats, microcomputed tomography, osteoporosis, osteoporosis induction, osteopenia, ovariectomized

## Abstract

**Background:** Osteoporosis is a systemic disease characterized by a progressive decrease in bone density and deterioration of the tissue’s microarchitecture. This results in greater structural fragility and a higher risk of fractures. Osteopenia represents the beginning of the process of decreasing bone density and, if left untreated, can lead to osteoporosis. The objective of this study was to validate an experimental model for establishing cases of decreased bone density that allows for the creation of different levels of severity of mineral loss and changes in bone microstructure. **Materials and Methods:** Twenty female Wistar rats, 12 weeks old and with a body weight ranging from 300 to 400 g, were used in this study. The animals were randomly distributed into five groups (*n* = 5 per group): a control group (CG), where the animals were not ovariectomized (OVX), and four experimental groups, where the animals were OVX and euthanized at different times: 30 days (G30), 40 days (G40), 60 days (G60), and 80 days (G80). The animals in the experimental groups underwent bilateral ovariectomy to induce mineral loss. The femurs were collected after the periods established for each group and analyzed using microcomputed tomography (μCT) to determine bone density and count the number of trabeculae. Furthermore, the T-score was calculated for each group. **Results:** There were significant differences in bone density when comparing all groups, with GC > G30 > G40 > G60 > G80. For the number of trabeculae, GC presented more trabeculae than all other groups. More trabeculae were also observed in G30 when compared to G40, G60, and G80; however, there were no differences between G40, G60, and G80. Regarding the calculation of the T-score by group, osteopenia was observed in G30 (T-score: −2.42) and osteoporosis was observed in G40, G60, and G80 (T-scores: −4.38, −6.34, and −7.71, respectively). **Conclusions:** The results demonstrate that ovariectomy induces progressive changes in bone structure, with the onset of osteopenia 30 days after ovariectomy and osteoporosis after 40 days in this experimental model. These results may aid future investigations that seek to focus on the specific treatment of osteopenia and/or osteoporosis.

## 1. Introduction

Osteoporosis is a metabolic bone condition characterized by decreased bone mineral density (BMD) and deterioration of the microarchitecture of bone tissue. This results in increased bone fragility and, consequently, greater susceptibility to fractures. In this sense, it is well established in the scientific literature that scores based on bone densitometry (e.g., T-score) are predictors of the level of impairment and risk for bone fractures [1,2]. This disorder affects millions of people worldwide, especially older individuals and postmenopausal women, where falling estrogen levels contribute significantly to decreased bone mineral density [3]. The causes of osteoporosis are multifactorial and include genetic, hormonal, nutritional, and environmental factors. Genetic predisposition can influence BMD, while hormones such as estrogen and testosterone play crucial roles in regulating bone metabolism. Nutritional deficiencies, especially in calcium and vitamin D, are equally relevant, since these nutrients are essential for the formation and maintenance of bone health [4,5]. In this sense, it is known that the first phase of bone mass reduction established as osteopenia tends to be progressive, leading to the establishment of osteoporosis [6]. In this sense, it is known that osteopenia is a critical window for intervention, as therapeutic strategies may prevent further bone loss and, consequently, prevent osteoporosis establishment and osteoporotic fractures [7].

The effects of osteoporosis are not limited to an increased risk of fractures but can also impact the quality of life of affected individuals. Fractures resulting from osteoporosis, especially in areas such as the spine, hip, femur, and wrist, are often accompanied by chronic pain, loss of mobility, and even serious complications that can lead to hospitalization and death in more severe cases [8]. Furthermore, osteoporosis can induce hypoventilation and an increased risk of falls, further amplifying the risk of subsequent fractures. In short, osteoporosis is a complex condition, with diverse etiologies and significant consequences for public health. A thorough understanding of its causes and effects is vital for the development of effective prevention and treatment strategies. Education about the importance of bone health and changes in lifestyle habits are essential to mitigate the impacts of osteoporosis on the population [3,4].

Osteoporosis treatment aims to increase bone mineral density, reduce the risk of fractures, and improve patients’ quality of life. Bisphosphonates, such as alendronate and risedronate, are often used as first-line therapy due to their effectiveness in reducing the incidence of vertebral and nonvertebral fractures [9]. Other options include selective estrogen receptor modulators (SERMs), such as raloxifene, which offer protection against fractures in postmenopausal women [10]. Therapy with denosumab, a monoclonal antibody that inhibits osteoclast formation, has also been shown to be effective in reducing the risk of fractures [11]. Additionally, calcium and vitamin D supplementation is recommended to optimize bone health and enhance the effects of medications [12]. Exercise strategies and lifestyle changes are complementary to pharmacological treatment, promoting mobility and muscle strength [13].

To understand the underlying pathophysiological mechanisms and develop new intervention strategies, experimental models are fundamental. The use of female rats as an animal model has proven effective in simulating osteoporosis, allowing for an evaluation of pharmacological and non-pharmacological interventions with adequate control of variables [14]. These animals offer advantages, such as a relatively short life cycle, easy handling, and bone characteristics that resemble those of humans, making them ideal for studies of bone loss induced by hormonal, nutritional, and environmental factors [15]. Osteoporosis induction in female rats can be achieved by ovariectomy, which mimics the loss of estrogen seen in menopausal women, or by diets deficient in calcium and vitamin D [5]. These models allow for a detailed investigation of the bone response to different treatments, such as anabolic and antiresorptive agents, and facilitate the understanding of the molecular mechanisms involved in bone remodeling [16].

Studies show that ovariectomy in female rats results in significant changes in bone microarchitecture, demonstrating the effectiveness of this experimental model in simulating postmenopausal osteoporosis [17,18]. Accurate determination of osteopenia and osteoporosis levels at different intervals after ovariectomy is critical to understanding the progression of hypoestrogenism-induced bone loss. It is worth noting that an assessment at different post-ovariectomy periods allows for the identification of critical time windows for therapeutic interventions, optimizing strategies for the prevention and treatment of osteoporosis. Therefore, establishing protocols that define the appropriate time for euthanasia of animals is essential for the standardization and reproducibility of results in osteometabolic research, allowing for the establishment of specific treatments for osteopenia and/or osteoporosis.

By studying osteoporosis in female rats, researchers can explore the interaction between genetic and environmental factors, contributing to the development of more effective preventive and therapeutic methods [14]. Therefore, the creation of experimental models in rats for the study of osteoporosis is crucial not only to elucidate the mechanisms of the disease but also to test new approaches that can be translated into clinical practice, improving the outcomes and quality of life of affected patients. In this sense, the objective of the present study was to validate an experimental model for establishing different levels of severity of bone density loss and microstructural changes through standardized microtomography, enabling the progressive characterization of osteopenia and osteoporosis in an ovariectomized model.

## 2. Materials and Methods

All procedures performed in this study were previously approved by the Animal Use Ethics Committee (CEUA) of Faculdade São Leopoldo Mandic (Campinas, Brazil) under protocol number 2024/11. The experiments fully complied with the provisions of Law No. 11,794 of 8 October 2008, Decree No. 6899 of 15 July 2009, and the standards established by the National Council for the Control of Animal Experimentation (CONCEA).

### 2.1. Animals and Group Formation

In the present study, twenty female Wistar rats (Rattus norvegicus), 12 weeks old and with a body weight ranging from 300 to 400 g, were used. The animals were kept under standard laboratory conditions, housed in plastic cages containing wood shavings, with light control in 12 h light/dark cycles, controlled temperature, adequate air exhaust, access to filtered and chlorinated water, and ad libitum feeding with specific industrialized feed (Nuvilab CR-1, Quimtia, Colombo, Brazil). Management was carried out in the Experimental Bioterium of the São Leopoldo Mandic Faculty. The animals were randomly distributed into four experimental groups, each consisting of five rats (*n* = 5 per group). Except for the control group (CG), all animals underwent bilateral ovariectomy to induce bone mineral loss, with the groups differentiated by the period previously established for euthanasia (Table 1).

### 2.2. Ovariectomy Surgery

The animals underwent surgery under general anesthesia, performed through intramuscular administration of ketamine hydrochloride (Ketalar injetável—Ache Laboratórios Farmacêuticos S.A., São Paulo, Brazil), at a dosage of 75 mg/kg of body weight, associated with the use of a muscle relaxant (xylazine hydrochloride, Vetnil, Louveira, Brazil) and veterinary sedative (Ropun—Bayer S.A., Leverkusen, Germany) at a dosage of 15 mL/kg. After the induction of anesthesia, trichotomy and disinfection procedures of the target area with a 2% chlorhexidine solution were performed.

**Figure 1:**
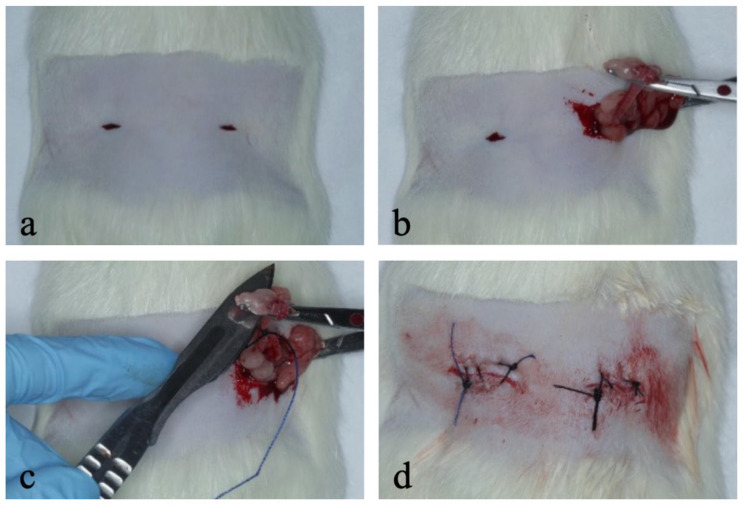
Ovariectomy surgical technique used in this study. (**a**) Ventral transverse incision of 0.5 cm to expose the ovaries; (**b**) fixation and mobilization of the ovary with Halstead forceps; (**c**) ligation with 4-0 Polyglycolic Acid-ABS suture for ovarian removal; (**d**) closing of incisions with sutures in the muscles and skin.

The bilateral ovariectomy surgical procedure was performed through 0.5 cm ventral transverse incisions, made with the aid of blade type 22, with the aim of exposing the ovaries (Figure 1a). The location of the bilateral incisions in the dorsal region was determined by palpation, associated with verification of the sacral tuberosity. After adequate exposure of the ovarian region, divulsion maneuvers were performed using Metzenbaum scissors to facilitate access to the ovarian structure [19]. For fixation of the ovary and uterine horn, a 12 cm straight Halstead clamp was used, positioned to allow mobilization and controlled removal of the ovary (Figure 1b). The ovarian removal itself was performed by applying a ligature using a 4-0 Polyglycolic Acid-ABS suture thread, (Biolint, Fortaleza, Brazil) to ensure vascular occlusion and safety of the excision with blade type 22 (Figure 1c). The procedure was closed in two stages. First, a simple suture of the dorsal muscles was performed, and then, the skin was sutured, ensuring the integrity of the abdominal cavity and adequate healing (Figure 1d).

### 2.3. Post-Operative Euthanasia and Specimen Processing

After surgery, the animals were housed in groups of four per cage and received a single dose of 600,000 IU of Benzetacil (Eurofarma Laboratórios SA, Ribeirão Preto, Brazil). All animals were kept in an environment with 12 h light and dark cycles, with the temperature controlled at 21 °C, in addition to an ad libitum diet. Acetaminophen, 110–305 mg/kg (Sanofi-Aventis Farmacêutica LTDA, Suzano, Brazil), was administered orally every 12 h as a post-surgical analgesic. The animals were euthanized according to the protocols established for the experimental groups, at zero, 30, 60, and 80 days, by means of an anesthetic overdose, using a saturated environment of 2% isoflurane (Syntec do Brasil, São Paulo, Brazil) followed by exsanguination through the coronary arteries. After collecting the femurs, they were skeletonized and immersed in a 10% formaldehyde solution. Subsequently, the animals were discarded in accordance with ethical and regulatory standards, being placed in white bags for incineration.

### 2.4. Microtomography Analysis

**Figure 2:**
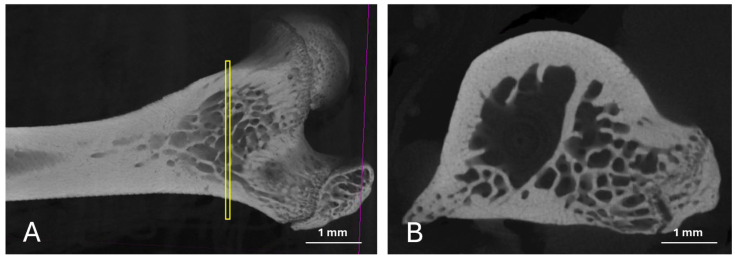
Representative images showing the sagittal cut position used to count trabeculae and assess density. (**A**) The position of the cut in each sample (yellow marks); (**B**) the cut obtained for the analysis.

All collected femurs were subjected to microtomography analysis using the SkyScan 1173 equipment (Bruker, Kontich, Belgium), with a voltage of 130 kV, current of 61 mA, and resolution of 6 μm, focusing on the proximal femoral epiphyses. Although the equipment allows for volumetric acquisition, in this study, we opted for a standardized two-dimensional (2D) approach. The region of interest (ROI) was defined based on three consecutive sagittal sections, standardized according to specific anatomical landmarks of the proximal femoral epiphysis (as indicated in Figure 2). These sections were obtained in the same relative position in all samples, with the aim of ensuring reproducibility and representativeness of the trabecular microarchitecture.

**Figure 3:**
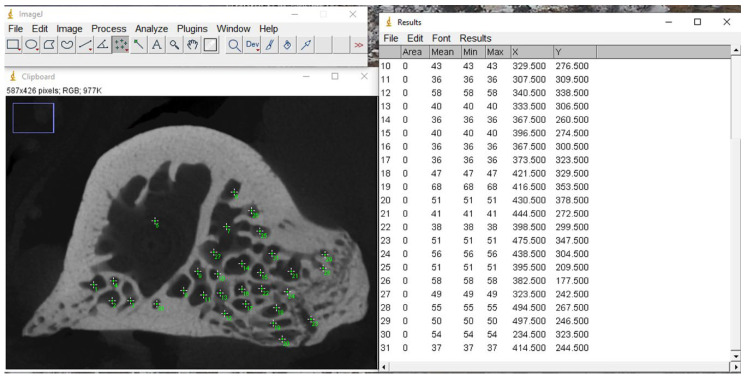
Image of the trabeculae number count using the ImageJ software.

After generating the images, the number of trabeculae was counted using the ImageJ software, version 1.53c (National Institutes of Health, Bethesda, Rockville, MD, USA), as shown in Figure 3.

**Figure 4:**
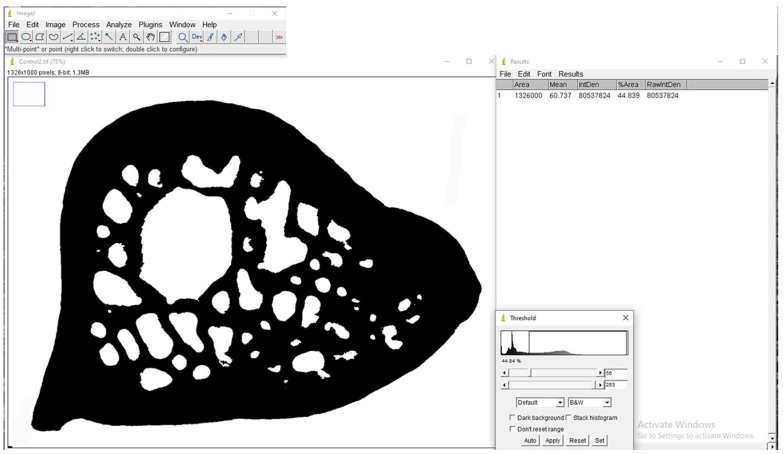
Representative image of the bone density percentage measurement using the ImageJ software.

Bone density percentage was also performed using the ImageJ software, version 1.53c (National Institutes of Health, Bethesda, USA), in this case, selecting the BoneJ plugin. The observer repeated the assessment three times during a 2-week interval. The measurement started with converting all DICOM data to 64-bit format and then determining the region of interest (ROI) using the freehand selection tool. The image preprocessing process used the Gaussian filter blur adjustment to smooth the objects and adjusted the threshold to brighten the edges of the trabeculae by examining the dark background. This converted the image into a binary form, and the trabeculae were separated from the non-trabeculae or bone marrow (Figure 4).

Next, the T-scores were calculated for each group. For this, the GC results were considered as standard, and the calculation was performed using an adapted formula [20]:

T-score = density − average GC/Standard Deviation of GC.

Based on the T-score results, the conditions were categorized, assigning a normal status when the T-score was above −1.0, a condition of osteopenia when the T-score varied between −1.0 and −2.5, and osteoporosis when the T-score was below −2.5 [1].

### 2.5. Statistical Analysis

The raw data collected were organized in a spreadsheet containing the following variables: number of trabeculae and bone density (%). For statistical analysis, data were subsequently transferred to the SigmaStat for Windows software, version 4.0 (Systat Software Inc., San Jose, CA, USA). For each of the parameters evaluated (number of trabeculae and density), the mean values, standard deviations, medians, minimum and maximum values, in addition to the respective 95% confidence intervals of the means were calculated. For the inferential statistical analysis, preliminary tests of normality and homogeneity of variances were initially applied to define the choice of the most appropriate statistical tests. In cases where the data presented normal distribution and homogeneous variances, one-way analysis of variance (ANOVA) was used. For non-normal distributions, the non-parametric Kruskal–Wallis test was used. When statistically significant differences were identified, multiple comparison tests were applied for detailed analysis between the groups. Both descriptive and inferential analyses were conducted using the SigmaStat for Windows software, version 4.0 (Systat Software Inc., Chicago, IL, USA), adopting a significance level of 5% for all statistical comparisons.

## 3. Results

**Figure 5:**
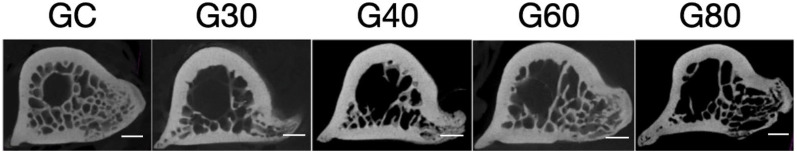
Representative image of the cuts of each group. GC: control group; G30: group at 30 days; G40: group at 40 days; G60: group at 60 days; G80: group at 80 days. The white scale bar in the images represents 1 mm.

The images of the sections obtained for each group showed a visible loss of bone mass, as demonstrated in Figure 5.

### 3.1. Bone Density

**Figure 6:**
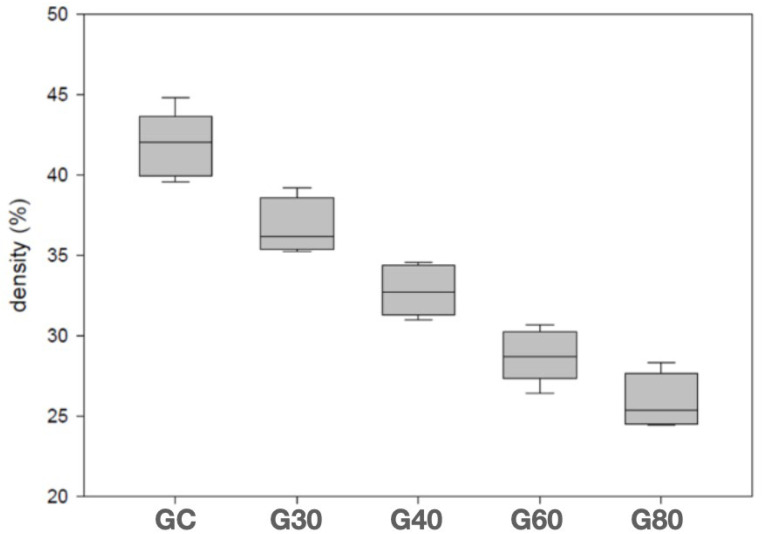
Box-plot graph (minimum value, 25% quartile, median, 75% quartile, and maximum value) of density in percentages (%).

Table 2 and the graph in Figure 6 show the bone density percentage results for all groups. A significant decrease in bone density over time was observed.

### 3.2. A Count of the Trabeculae Number

**Figure 7:**
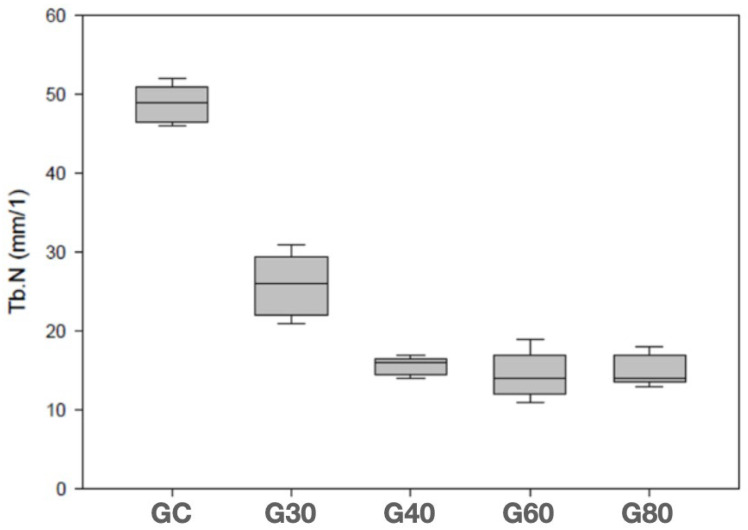
Box-plot graph (minimum value, 25% quartile, median, 75% quartile, and maximum value) of trabeculae number.

Table 3 shows the results of the number of trabeculae for all groups. It was possible to observe a progressive decrease in the number of trabeculae up to 40 days. Figure 7 shows a graph with the trabeculae number distribution between the groups.

### 3.3. T-Score and Categorization

Table 4 illustrates the results of the T-scores and also shows the inference of the determination of the normality, osteopenia, and osteoporosis framework based on a correlation with the T-scores. Based on the categorization system proposed in the method of this study (see Section 2.4), it was possible to observe the establishment of the osteopenia framework in 30 days and the osteoporosis framework after 40 days.

## 4. Discussion

Osteoporosis is a metabolic bone disease characterized by reduced bone mass and deterioration of the microarchitecture of bone tissue, resulting in increased fragility and susceptibility to fractures [4,5], which represents a public health problem, since it affects one in ten people over 50 years of age in the USA [21]. In this context, among the experimental models used to study osteoporosis, ovariectomy in female rats has proven to be one of the most reliable, as it induces bone changes similar to those observed in postmenopausal women [15]. Surgical removal of the ovaries results in an abrupt reduction in estrogen levels, causing an imbalance between bone resorption and formation, which culminates in progressive bone loss and increased skeletal fragility [17,18]. This model has been widely accepted in osteoprotective therapy research due to its reliability and reproducibility [16], which led our group to select the ovariectomy procedure to generate bone density reduction in the present study.

A clear aspect in science is the standardization of experimental protocols to ensure the reproducibility and reliability of the data obtained [16]. For the type of experimental model proposed in the present study, the definition of specific periods for euthanasia of animals allows for an evaluation of the progression of bone loss and the response to the treatments investigated. This is particularly relevant to differentiate progressive stages of bone loss, contributing to a better understanding of the different degrees of osteoporosis [2]. The time of euthanasia in experimental models directly influences the magnitude of the decrease in bone density, making it possible to correlate different evaluation periods with the degrees of severity of osteoporosis. Studies show that bone loss tends to increase progressively over time, being more evident in more advanced phases of the experimental model, which reinforces the need for well-established criteria for classifying and analyzing the results obtained [5]. Furthermore, variables such as age, lineage, and diet of the animals must be strictly controlled to avoid interference in the results [14].

Studies show that ovariectomy leads to significant changes in the bone structure of animals, including a decrease in the number of trabeculae, increased porosity, and reduced bone mineral density [5]. These changes are fundamental for the investigation of new pharmacological and non-pharmacological therapies for osteoporosis, allowing for an evaluation of the efficacy of bioactive compounds, nutritional supplements, and physical interventions in the prevention of bone loss [14,15]. Therefore, it is reasonable to consider the importance of the present study, which focused on determining the establishment of different levels of severity of mineral loss and changes in bone microstructure according to the time elapsed since an ovariectomy procedure in one of the animals most used in scientific research (Wistar rats). In this sense, it is important to state that the ovariectomized rat model is considered a gold standard model for postmenopausal osteoporosis research and recognized by FDA as the most appropriate preclinical model [22].

The analysis of the results obtained by the present study reinforces the validity of the experimental model of ovariectomy in female Wistar rats for the study of osteoporosis. According to Yousefzadeh et al., 2019 [23], Wistar and Sprague–Dawley rats are the most common breeds for studying osteoporosis and show similar responses to ovariectomy. Regarding changes in bone density, a gradual and significant decrease was observed over time after ovariectomy (see Table 2). The change in bone microstructure generated by ovariectomy resulted in a significant decrease in the number of trabeculae between the control situation (without ovariectomy) compared to all other times. There was also a significant decrease in the number of trabeculae between 30 days after ovariectomy and 40, 60, and 80 days but not between 40 days and 60 and 80 days (see Table 3). These results of progressive loss are in accordance with previous studies in the literature [24] and may be correlated with higher levels of fracture risk over time, although no episodes of bone fracture occurred in the present study.

The use of the T-score is widely considered as a tool for categorizing bone density status [1,2]. In this sense, defining standards for the classification of osteoporosis is essential to ensure accuracy in the diagnosis and assessment of the severity of the disease. The distinction between normality, osteopenia, and osteoporosis is based on T-score values obtained through bone densitometry, with osteopenia being defined by a T-score between −1 and −2.5, while osteoporosis is characterized by values below −2.5 [2]. This differentiation is crucial for choosing therapeutic approaches, since the progression of bone loss may require different interventions, including calcium and vitamin D supplementation; pharmacological therapies, such as bisphosphonates; and rehabilitation strategies to prevent fractures [4]. Therefore, the T-score results presented in this study, calculated using the adapted formula (see Item 2.4), represent an important understanding for standardizing changes in bone density in this experimental model. Since the 30-day post-ovariectomy period showed a T-score of −2.42, a case of osteopenia can be inferred. However, the T-scores at 40, 60, and 80 days (−4.38, −6.34, and −7.71, respectively) represent established osteoporosis. Therefore, future studies for the treatment of decreased bone density will be able to use the results of the present study to select the severity of the osteoporotic disease to be addressed. In this scope, interventional studies in Wistar rats could use the data of the present study to verify the performance of different therapeutical agents in situations of osteopenia (G30 of the present study) or different magnitude osteoporosis (G40, G60, and G80 of the present study). Even though a perfect translation of human T-scores to rat T-scores is tricky, there is an antecedent in the literature [25]. In any case, the current study’s T-score levels’ gradual decline over time clearly shows that the condition being evaluated has gotten worse.

Taking as a basis the specific comparison between normality (GC) and the presence of established osteoporosis (G40), a decrease of 21.57% in bone density can be observed (41.852 ± 2.061% in GC versus 32.824 ± 1.583% in G40) and a decrease of 68.03% in the number of bone trabeculae (48.8 ± 2.38% in GC versus 15.6 ± 1.14% in G40), which denotes a clear tendency towards bone fragility. Still, regarding the specific comparison between GC and G40, based on the T-score results, it can be inferred that there was a jump of two conditions: as a picture of normality was left, the osteopenia picture was overcome (G30), and a picture of effective osteoporosis was reached in G40 (see Table 4). However, it is important to consider that in the present study, no mechanical evaluation was performed to assess bone fragility. According to Cosman et al. [1], a normal condition is determined when the T-score is above −1.0, osteopenia is determined when the T-score varies between −1.0 and −2.5, and osteoporosis is determined when the T-score is below −2.5, with any condition with a T-score below −2.5 and associated with at least one fracture being categorized as severe osteoporosis. In this sense, as no episode of bone fracture was observed in the present study, it is understood that the establishment of severe osteoporosis cannot be inferred, despite G80 having represented a significant decrease in bone density in the order of 38.00% compared to the control situation (41.852 ± 2.061% in GC versus 25.946 ± 1.671% in G80). However, this may be related to limitations of the experimental model of the present study, including those related to ethical issues in animal research, where rats were kept in plastic cages with minimal access to trauma conditions that could induce bone fractures. Despite this, the G80 results give us a glimpse of the establishment of a very critical loss of bone mineral density, which may be useful for future investigations that focus on the treatment of severe bone mass losses, therefore opening the possibility for an eventual translation to the clinical conditions of severe osteoporosis.

Our group believes that order and classification form the beginning of mastery and that, therefore, the validation of the experimental model of ovariectomy in female Wistar rats reinforces its relevance in osteoporosis research, enabling significant advances in the understanding of the pathophysiology of the disease and in the development of new therapeutic approaches for specific severities (i.e., normality, osteopenia, and osteoporosis). However, it is worth considering that the present study focused on a specific type of experimental model (i.e., induction of mineral bone loss through ovariectomy in female Wistar rats that were 12 weeks old and with a body weight ranging from 300 to 400 g) and, therefore, cannot be extrapolated to different models. Moreover, it is important to state that we did not use a simulated surgery for the control group, which might have subtle effects on bone turnover. Future studies should continue to explore this model, improving experimental protocols and investigating new therapeutic targets to mitigate the deleterious effects of osteopenia/osteoporosis through bioengineering.

Furthermore, it is important to highlight that despite advances in three-dimensional imaging techniques, a two-dimensional (2D) analysis still represents a valid and widely used tool to assess changes in bone density and trabecular microarchitecture, especially in experimental models with limited resources. Several studies demonstrate that depending on the research objective, a 2D analysis can provide relevant and consistent data over time, being effective in characterizing different stages of bone loss [26,27,28]. According to Bouxsein et al. (2010) [26], parameters obtained through 2D images, such as trabecular thickness, the number of trabeculae, and trabecular separation, present good correlations with three-dimensional measurements obtained by microtomography, particularly when there is standardization in sampling and image processing. Furthermore, other authors reinforce that a 2D analysis can be applied at different periods after ovariectomy to detect progressions in mineral loss and structural changes [27,28]. Therefore, the results obtained in the present research, even though they are based on a 2D evaluation, are representative of the progressive bone loss process and can be used with confidence in the characterization of osteopenia and osteoporosis at different stages.

A methodological limitation recognized in this study is the use of two-dimensional images for bone microstructural analysis instead of complete three-dimensional reconstructions. However, we sought to mitigate this factor through the standardized selection of multiple representative slices and systematic repetitions of measurements with control of intraobserver variability.

## 5. Conclusions

The results demonstrate that ovariectomy in female Wistar rats induces progressive changes in bone structure, with the onset of osteopenia 30 days after ovariectomy and progressive osteoporosis after 40 days. These results may aid future research that seeks to focus on the specific treatment of osteopenia and/or osteoporosis.

## Figures and Tables

**Table 1:** Characterization of groups.

Group	Description	Procedure	Time Until Euthanasia
GC	Control	Non-ovariectomized animals	Not applicable
G30	Ovariectomized	Euthanasia after ovariectomy	30 days
G40	Ovariectomized	Euthanasia after ovariectomy	40 days
G60	Ovariectomized	Euthanasia after ovariectomy	60 days
G80	Ovariectomized	Euthanasia after ovariectomy	80 days

**Table 2:** Mean and standard deviation (%) of bone density measured for each group.

	GC	G30	G40	G60	G80
Mean	41.852 ^A^	36.844 ^B^	32.824 ^C^	28.782 ^D^	25.946 ^E^
SD	2.061	1.716	1.583	1.623	1.671

GC: control group; G30: group at 30 days; G40: group at 40 days; G60: group at 60 days; G80: group at 80 days. Different letters represent a statistically significant difference between groups.

**Table 3:** Mean and standard deviation of the count of the trabeculae number.

	GC	G30	G40	G60	G80
Mean	48.8 ^A^	25.8 ^B^	15.6 ^C^	14.4 ^C^	15 ^C^
SD	2.38	3.96	1.14	2.96	2

SD: standard deviation; GC: control group; G30: group at 30 days; G40: group at 40 days; G60: group at 60 days; G80: group at 80 days. Different letters represent a statistically significant difference between groups.

**Table 4:** T-scores and categorization (determination of normality, osteopenia, and osteoporosis).

	GC	G30	G40	G60	G80
T-score	0	−2.42	−4.38	−6.34	−7.71
Categ.	normality	osteopenia	osteoporosis	osteoporosis	osteoporosis

GC: control group; G30: group at 30 days; G40: group at 40 days; G60: group at 60 days; G80: group at 80 days.

## Data Availability

All relevant data are contained within this paper.

## References

[1] Cosman F., de Beur S.J., LeBoff M.S., Lewiecki E.M., Tanner B., Randall S., Lindsay R. (2014). National Osteoporosis Foundation. Clinician’s Guide to Prevention and Treatment of Osteoporosis. Osteoporos. Int..

[2] Kanis J.A., Burlet N., Cooper C., Delmas P.D., Reginster J.Y., Borgstrom F., Rizzoli R. (2008). European guidance for the diagnosis and management of osteoporosis in postmenopausal women. Osteoporos. Int..

[3] Hauselmann H.J., Rizzoli R. (2003). A comprehensive review of treatments for postmenopausal osteoporosis. Osteoporos. Int..

[4] Compston J.E., McGowan B.M., Bowring C. (2019). Osteoporosis and fracture risk: The role of bone turnover. Osteoporos. Int..

[5] Rachner T.D., Khosla S., Hofbauer L.C. (2011). Osteoporosis: Now and the future. Lancet.

[6] Netelenbos J.C., Lems W.F., Geusens P.P., Verhaar H.J., Boermans A.J., Boomsma M.M., Mulder P.G., Papapoulos S.E. (2009). Spine radiographs to improve the identification of women at high risk for fractures. Osteoporos. Int..

[7] Karaguzel G., Holick M.F. (2010). Diagnosis and treatment of osteopenia. Rev. Endocr. Metab. Disord..

[8] Johnell O., Kanis J.A. (2006). An estimate of the worldwide prevalence and disability associated with osteoporotic fractures. Osteoporos. Int..

[9] Alwahhabi B.K., Alsuwaine B.A. (2017). Long-term use of bisphosphonates in osteoporosis. Saudi Med. J..

[10] Cummings S.R., Eckert S., Krueger K.A., Grady D., Powles T.J., Cauley J.A., Norton L., Nickelsen T., Bjarnason N.H., Morrow M. (1999). The effect of raloxifene on risk of breast cancer in postmenopausal women: Results from the MORE randomized trial. Multiple Outcomes of Raloxifene Evaluation. JAMA.

[11] Zaheer S., LeBoff M., Lewiecki E.M. (2015). Denosumab for the treatment of osteoporosis. Expert. Opin. Drug Metab. Toxicol..

[12] Weaver C.M., Gordon C.M., Janz K.F., Kalkwarf H.J., Lappe J.M., Lewis R., O’Karma M., Wallace T.C., Zemel B.S. (2016). The National Osteoporosis Foundation’s position statement on peak bone mass development and lifestyle factors: A systematic review and implementation recommendations. Osteoporos. Int..

[13] Jessup J.V., Horne C., Vishen R.K., Wheeler D. (2003). Effects of Exercise on bone density, Balance, and Self-Efficacy in Older Women. Biol. Res. Nurs..

[14] Frost H.M., Jee W.S. (1992). On the rat model of human osteopenias and osteoporoses. Bone Miner..

[15] Mathavan N., Tägil M., Isaksson H. (2017). Do osteoporotic fractures constitute a greater recalcitrant challenge for skeletal regeneration? Investigating the efficacy of BMP-7 and zoledronate treatment of diaphyseal fractures in an open fracture osteoporotic rat model. Osteoporos. Int..

[16] Baird K.S., McCarthy H.S. (2018). The importance of animal models in osteoporosis research. Bone Rep..

[17] Cheung W.H., Lee K.M., Kung A.W. (2014). Development of an osteoporotic animal model. Int. J. Mol. Sci..

[18] Cheung W.H., Yu P.H., Lu W.W. (2014). Osteoporosis models for evaluation of treatment regimens. J. Orthop. Translat..

[19] Fischer L., Torres-Chávez K.E., Clemente-Napimoga J.T., Jorge D., Arsati F., de Arruda Veiga M.C., Tambeli C.H. (2008). The influence of sex and ovarian hormones on temporomandibular joint nociception in rats. J. Pain..

[20] Lewiecki E.M., Borges J.L. (2006). Bone density testing in clinical practice. Arq. Bras. Endocrinol. Metabol..

[21] Wright N.C., Looker A.C., Saag K.G., Curtis J.R., Delzell E.S., Randall S., Dawson-Hughes B. (2014). The recent prevalence of osteoporosis and low bone mass in the United States based on bone mineral density at the femoral neck or lumbar spine. J. Bone Miner. Res..

[22] U.S Department of Health and Human Services—Food and Drug Administration Osteoporosis: Nonclinical Evaluation of Drugs Intended for Treatment—Guidance for Industry..

[23] Yousefzadeh N., Kashfi K., Jeddi S., Ghasemi A. (2020). Ovariectomized rat model of osteoporosis: A practical guide. EXCLI J..

[24] Fang J., Yang L., Zhang R., Zhu X., Wang P. (2015). Are there differences between Sprague-Dawley and Wistar rats in long-term effects of ovariectomy as a model for postmenopausal osteoporosis?. Int. J. Clin. Exp. Pathol..

[25] Brzóska M.M., Moniuszko-Jakoniuk J. (2004). Low-level lifetime exposure to cadmium decreases skeletal mineralization and enhances bone loss in aged rats. Bone.

[26] Bouxsein M.L., Boyd S.K., Christiansen B.A., Guldberg R.E., Jepsen K.J., Müller R. (2010). Guidelines for assessment of bone microstructure in rodents using micro-computed tomography. J. Bone Miner. Res..

[27] Wang L., You X., Zhang L., Zhang C., Zou W. (2022). Mechanical regulation of bone remodeling. Bone Res..

[28] Tao Z., Li T.L., Yang M., Xu H.G. (2022). Silibinin Can Promote Bone Regeneration of Selenium Hydrogel by Reducing the Oxidative Stress Pathway in Ovariectomized Rats. Calcif. Tissue Int..

